# NDRG1 is a prognostic biomarker in breast cancer and breast cancer brain metastasis

**DOI:** 10.1002/2056-4538.12364

**Published:** 2024-02-05

**Authors:** Vaibhavi Joshi, Andrew Stacey, Yufan Feng, Priyakshi Kalita‐de Croft, Pascal HG Duijf, Peter T Simpson, Sunil R Lakhani, Amy E McCart Reed

**Affiliations:** ^1^ UQ Centre for Clinical Research, Faculty of Medicine The University of Queensland Brisbane Australia; ^2^ Centre for Cancer Biology, Clinical and Health Sciences University of South Australia & SA Pathology Adelaide Australia; ^3^ Pathology Queensland The Royal Brisbane and Women's Hospital Brisbane Australia

**Keywords:** breast cancer, brain metastasis, biomarker, NDRG1, prognosis, Goldilocks

## Abstract

Brain metastases are secondary brain tumours characterised by their aggressive nature and poor prognosis. Breast cancer is one of the most common primary tumours in women to spread to the brain. A lack of biomarkers predicting likely spread to the brain and limited therapeutic interventions represents major areas of clinical unmet need. We investigated N‐myc downregulated gene‐1 (NDRG1) as a clinically relevant biomarker in breast cancer brain metastasis patients. NDRG1 expression was investigated using immunohistochemistry on tissue microarrays of two clinical cohorts: (i) brain metastatic breast cancers (*n* = 48) and brain metastases (*n* = 64; including a subset of 39 patient‐matched breast and brain metastasis cases) and (ii) unselected primary breast cancers (*n* = 336). NDRG1 was highly expressed in breast‐to‐brain metastases, as well as in high‐grade primary breast cancers. High NDRG1 expression and also an absence of expression were associated with worse survival outcomes in both breast cancer and breast cancer brain metastasis patients. This establishes NDRG1 as a ‘Goldilocks’ protein, where too much or too little has a negative effect on survival. We pose that this accounts for its previous categorisation as both tumour suppressor and oncoprotein. Additionally, a shift in NDRG1 localisation with a gain of nuclear expression was seen at the brain metastasis stage. Significant survival benefit in cases expressing cytoplasmic NDRG1 was observed, whereas NDRG1 localisation in the nucleus showed a clear association with poorer survival. *In vitro* analyses revealed that hypoxic stress significantly elevated NDRG1 expression and resulted in its nuclear localisation. Our findings suggest NDRG1 expression and subcellular localisation are clinically relevant biomarkers for poor prognosis in breast cancer and breast cancer brain metastasis.

## Introduction

Breast cancer (BC) is one of the most prevalent cancers in the world, the most common in women and the cause of approximately 685,000 annual deaths worldwide [1]. BC is highly heterogeneous with a range of distinct molecular and biological features. The clinical classification of BC is based on the expression of the oestrogen receptor (ER), progesterone receptor (PR) and human epidermal growth factor receptor 2 (HER2), resulting in groups of hormone receptor‐positive (ER and PR positive), HER2‐positive and triple‐negative BC [triple‐negative breast cancer (TNBC); lacking ER and PR and without HER2 amplification] [2, 3, 4]. BC deaths typically result from metastatic spread [5], and BC cases without metastasis (‘early stage’) are considered treatable and eligible for curative therapy, whereas metastatic BC is often considered incurable [6]. Up to 30% of patients with metastatic BC present with brain metastases (BrMs), resulting in a poor prognosis (median survival range of 7–17 months) [7]. HER2‐positive and TNBC patients are at a higher risk of developing BrMs. BrMs are aggressive, have limited treatment options and severely affect patient quality of life. A lack of treatment options is one of the major challenges in the clinical management of BrM, highlighting the requirement for developing better therapeutic approaches [8]. As such, prevention, or early detection of BrM, where surgery may be beneficial, is important for improving outcomes for patients. Indeed, predicting which patients may progress to a BrM and could, therefore, be monitored more closely is clinically important. In this study, we consider N‐myc downstream‐regulated gene 1 (NDRG1) as a potential biomarker for risk prediction of BrM progression.

NDRG1 has been variably reported as a metastasis suppressor, a biomarker of poor outcome and a facilitator of disease progression in many cancers [9, 10]. A member of the NDRG family, NDRG1, is a 43 kDa protein with multiple reported isoforms, and its expression is associated with cellular processes such as differentiation, stress response and cell growth [11]. NDRG1 has been shown to suppress metastasis in pancreatic, ovarian and colorectal cancers, whereas in lung, cervical and hepatocellular cancers, NDRG1 has been associated with tumour progression [9]. There are limited data on the role NDRG1 plays in BC progression. Villodre *et al* demonstrated that NDRG1 is an independent factor of worse prognosis in pre‐treated inflammatory BC patients [12] and reported elevated NDRG1 expression to be linked with poorer clinical outcomes in the aggressive phenotypes of BC (HER2+, ER− or TNBC). Additionally, using publicly available datasets, *NDRG1* expression was shown to be higher in BrM compared with the corresponding primary tumours. They went on to show that over‐expression of NDRG1 drives brain metastatic progression in mouse models [13]. We present a study investigating the expression of NDRG1 in a clinical cohort of matched breast and metastatic brain tumours, and demonstrate the importance of NDRG1 subcellular localisation in prognostication.

## Materials and methods

### Clinical samples

The study has ethical approval from the Royal Brisbane Women's Hospital (RBWH) (2005/022) and The University of Queensland (2005000785) for the use of clinical data and samples from BC and BrM patients.

Two independent clinical cohorts were studied: (1) the Queensland Follow‐Up (QFU) cohort, which is comprised of formalin‐fixed paraffin‐embedded (FFPE) breast tumours from 336 patients that underwent breast tumour resection at the RBWH between 1987 and 1994, along with their long‐term clinical follow‐up information (median: 13.5 years; range: 0.2–42 years) and (2) the Queensland breast cancer brain metastasis (QBBM) cohort, composed of archival FFPE primary BC from brain metastatic patients (*n* = 48) and BrM samples (*n* = 64), including 39 matched BC‐BrM pairs, treated between the years 2000 and 2018. Pathology reports, clinical diagnostic information and survival data were obtained from Pathology Queensland, Queensland Health and the Queensland Cancer Registry. The clinicopathological information, such as the histological type, grade and stage, was curated. Tumours were sampled in tissue microarrays as 0.6 mm (QFU) or 1 mm (QBBM) cores for immunohistochemistry analysis. Complete treatment information is limited in these cohorts because of the historical nature of the dataset. Survival analysis was assessed as BC‐specific survival (BCSS)—calculated from the time of BC diagnosis to the last follow‐up, and BrM specific survival (BrMSS)—calculated from the time of BrM resection to the last known follow‐up.

### Immunohistochemistry

Antigen retrieval was performed on 4 μm sections with heat‐induced epitope retrieval in a decloaking chamber (Nexgen, Biocare Medical, Concord, CA, USA) with sodium citrate (0.01 m, pH 6.0), 110 °C for 20 min. The MACH1 Universal HRP Detection Kit (Biocare Medical, LLC.) was used for visualisation. The non‐specific background staining was blocked using the MACH1 sniper blocking reagent (BioCare Medical) for 30 min, followed by overnight incubation with the NDRG1 antibody (1:250; Abcam, EPR5593). The slides were treated with MACH1 secondary antibody conjugated with universal horseradish peroxidase (HRP polymer) for 30 min at room temperature and were counterstained with haematoxylin.

The scoring for both patient cohorts was performed with a pathologist (AS). A positive stain was defined as >1% of cells displaying unequivocal staining, whereas a negative finding was defined as <1% positivity. The intensity of stained cells was scored as weak (1+), moderate (2+) and strong (3+). Additionally, the subcellular localisation pattern of protein expression was documented as either cytoplasmic or cytoplasmic + membrane (grouped under non‐nuclear staining); or nuclear and cytoplasmic ± membrane (grouped under nuclear staining).

### Cell lines and culture

Human BC cell lines MDA‐MB‐231 and BT‐474 were obtained from the American Type Culture Collection. Brain seeking BC cell lines MDA‐MB‐231.Br and BT‐474.Br were kindly provided by Dr. Dihua Yu (MD Anderson Cancer Center, Houston, TX, USA). MDA‐MB‐231 and MDA‐MB‐231.Br were maintained in DMEM (Gibco, New York, USA; Cat# 1195065), BT‐474 and BT‐474.Br were maintained in RPMI (Gibco; Cat# 11875093) and all were supplemented with 10% foetal bovine serum (Gibco/Invitrogen, New York, USA; Cat#10099141) and antibiotic/antimycotic (Ab/Am; 1×, Gibco/Invitrogen; Cat# 11360).

### Immunofluorescence and imaging

Cells were seeded on autoclaved glass coverslips at a density of 0.1–0.3 × 10^4^ cells/24 wells. Cells were cultured either in a standard incubator under normoxic conditions (21% O_2_) or, in hypoxic conditions (3.8% O_2_) in a O_2_‐controlled incubator (Cell IQ™ CO_2_/O_2_ Incubator, Panasonic) for 24 h. Cells were fixed with 4% paraformaldehyde, washed with ice cold 1× PBS and permeabilised with 0.5% Triton‐X for 5 min. Cells were blocked with 2% bovine serum albumin (BSA) in PBS for 30 min before overnight incubation with a primary antibody cocktail for NDRG1 (1:1000; Abcam, EPR5593) and Beta‐catenin (1:400; BD biosciences, 610153) in a humidified chamber. Cells were washed with PBS and then incubated with secondary antibodies [goat anti‐rabbit IgG (H + L) Alexa Flour 594, Invitrogen; goat anti‐mouse IgG Alexa Flour 488, Invitrogen] before confocal microscopy imaging. The nuclear localisation of NDRG1 was quantified using the Zeiss Zen 3.5 (blue edition) software. Regions of interest (ROI, *n* = 14) were selected, and the mean fluorescent intensity of NDRG1 within the ROI was measured and normalised to the area of the ROI. Orthogonal images from Z‐stack files were developed using the Fiji‐ImageJ software [14].

### Western blotting

Extracted protein samples were quantified using the Pierce BCA Protein Assay Kit (ThermoScientific, Rockford, IL, USA; Cat#23225). A total of 20 μg of each sample was resolved on 4–12% bis‐tris SDS‐PAGE gels (Bolt, Invitrogen, ThermoScientific). The separated proteins were transferred onto a PVDF membrane (polyvinylidene fluoride; Millipore, MA, USA; Cat# IPVH00010) and were blocked for 1 h with 5% BSA solution in TBS with 0.1% Tween‐20 (TBST) (Sigma Life Science, MO, USA; Cat# P2287). Membranes were probed with either NDRG1 (1:10,000 Abcam, Cambridge, UK, EPR5593) or Beta actin (1:10,000; Cell signalling, Danvers, MA, USA; Cat# 8H10D10) at 4 °C, washed thrice with TBST, incubated for 1 h with HRP linked‐secondary antibody (anti‐mouse/anti‐rabbit) and visualised with SuperSignal ECL HRP substrate (West PicoPlus, Thermo Scientific) and the Chemidoc MP Imaging System (Bio Rad, South Granville, NSW, Australia).

### *NDRG1* copy number and expression in primary and brain metastatic breast cancers

In BC samples from The Cancer Genome Atlas (TCGA) [15] and the Metastatic Breast Cancer project (https://mbcproject.org) [16], *NDRG1* DNA copy number (determined using GISTIC, where gain is three copies and amplification is >3) was compared with *NDRG1* mRNA expression levels (RNAseq RSEM). *NDRG1* (Ensembl ID ENSG00000104419) expression in primary BCs (*n* = 45) was also compared with the expression in matched BrMs (*n* = 45; total *n* = 90) in previously published samples [17]. Exome‐capture RNA sequencing data were batch‐corrected, and *NDRG1* expression values were represented as log_2_‐transformed trimmed M of means (TMM) normalised counts per million (CPM) [i.e. log_2_(TMM‐CPM + 1)]. Since data were normally distributed, as determined using Kolmogorov–Smirnov tests, a two‐sided paired Student *t* test was applied to assess whether *NDRG1* expression was statistically significantly different in primary compared with matched brain metastatic BCs. Additionally, *NDRG1* (probe ID ‘g5174656_3p_s_at’) expression levels were assessed in an independent dataset comprising 19 HER2+ nonmetastatic primary BCs and 19 unmatched HER2+ BC BrMs [18]. Data were not normally distributed (*p =* 0.0303; Kolmogorov–Smirnov test). Hence, to determine whether expression levels were statistically significantly different between the groups, a two‐sided Mann–Whitney *U* test was applied.

### Survival analyses of *NDRG1* transcript levels in breast cancer

Kaplan–Meier recurrence‐free survival analysis in publicly available data from BC patients was performed using the Kaplan–Meier plotter (https://kmplot.com) [19]. The median expression of *NDRG1* (probe ID ‘200632_s_at’) was used as the cut‐off between the *NDRG1*‐low and *NDRG1*‐high expressing patients.

### Gene expression signatures in breast cancer

Single‐sample gene set enrichment analysis scores were determined using hypoxia and angiogenesis gene expression signatures in BC samples of the TCGA RNAseq dataset. Hypoxia signatures: ‘Winter’: 99‐gene expression signature [20]; ‘West’: 26‐gene signature [21]; ‘Sorensen’: 27‐gene signature [22]; ‘Seigneuric’ [23]; ‘Ragnum’: 32‐gene signature [24]; ‘Hu’: 13‐gene signature [25]; ‘Elvidge’ [26]; ‘Buffa’: 51‐gene signature [27]; and ‘HIF‐induced gene expression’: ‘Regulation of gene expression by hypoxia inducible factor’ signature from Reactome [28]. Angiogenesis signatures: ‘Masiero’: 43‐gene signature [29]; ‘integrins in angiogenesis’, were from PathCards (https://pathcards.genecards.org) [30]. These signature scores were compared with the *NDRG1* expression levels in each sample using Spearman's rank correlations.

### Statistical analysis

Raw data from each experiment were analysed using GraphPad Prism (v9.0). For the multivariate survival analysis, we utilised the *Survivalanalysis* package in R [31]. The statistical tests performed for each experiment and the *p* value are indicated in the respective figure legends. A *p* value was considered significant if *p* < 0.05.

## Results

### NDRG1 expression differs across breast cancer subtypes

**Figure 1:**
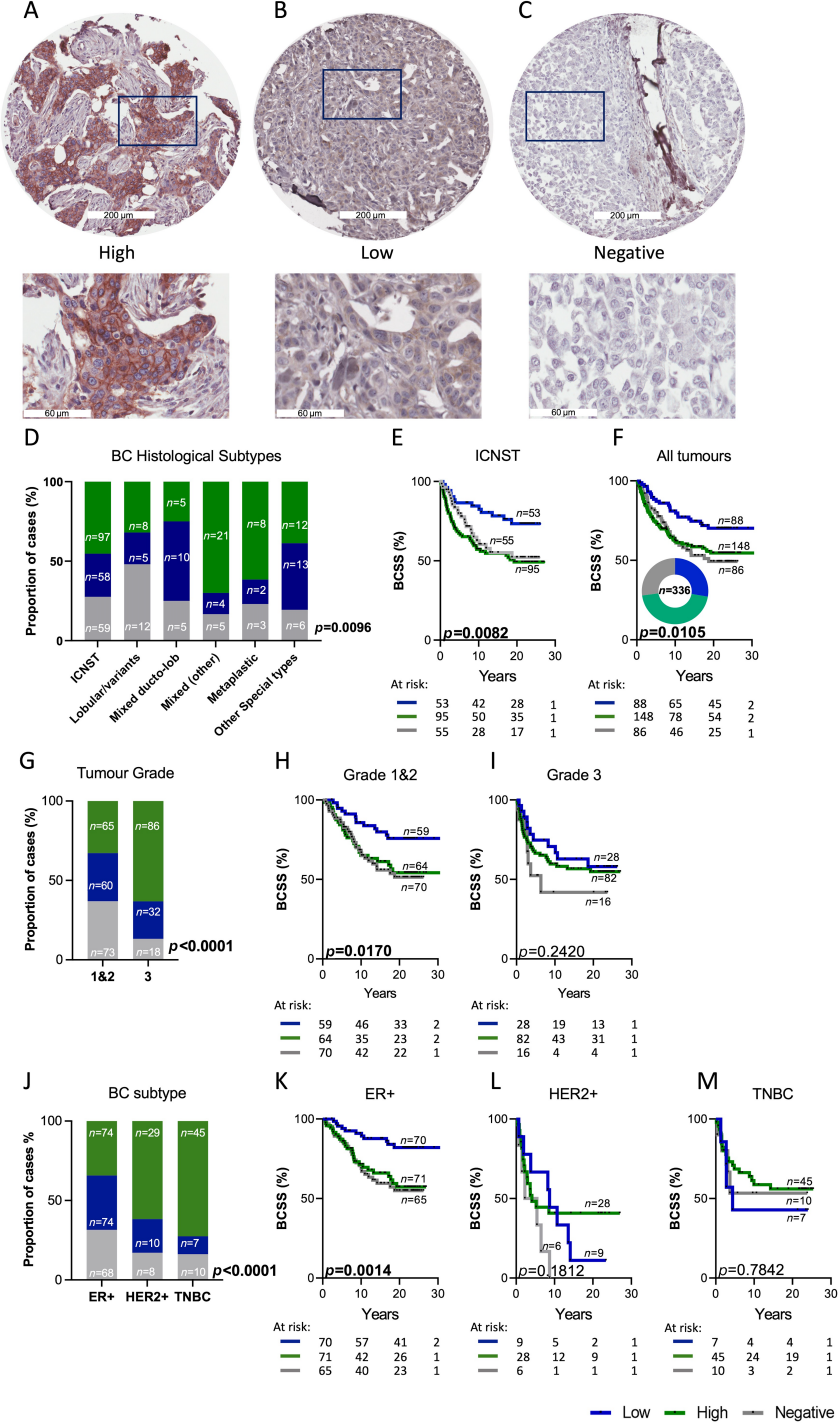
NDRG1 expression in breast cancer. Representative IHC images of breast tumour cores stained with NDRG1 stratified as (A) high, (B) low and (C) negative for NDRG1 expression. (D) Chi‐square analysis of proportions of breast cancer cases with low, high and negative NDRG1 expressions compared across the histological subtypes. KM analysis of NDRG1 high, low and negative tumours for BCSS in (E) IC‐NST and (F) all tumours. (G) Chi‐square analysis of proportions of breast cancer cases with high, low, and negative NDRG1 expressions compared across tumour grades. KM analysis of NDRG1 expression and BCSS in (H) grades 1 and 2 and (I) grade 3 tumours. (J) Chi‐square analysis of proportions of breast cancer cases with high, low and negative NDRG1 expressions compared across breast cancer clinical subtypes. KM analysis of NDRG1 expression and BCSS in (K) HER2+, (L) ER+ and (M) TNBC cases. BCSS, Breast cancer‐specific survival; ER+, oestrogen receptor; HER2+, Human epidermal growth factor receptor 2; IHC, immunohistochemistry; KM, Kaplan–Meier; TNBC, triple‐negative breast cancer.

To investigate NDRG1 expression in BC, we applied immunohistochemistry in an unselected, archival BC cohort of 336 patients with >25 years of clinical follow‐up. Table 1 summarises the clinicopathological characteristics of the tumours. NDRG1 expression was observed in many of the cases (*n* = 239/336; 72%), and cases were stratified into groups based on the intensity of expression (NDRG1 high; 2+ and 3+, Figure 1A); low (1+, Figure 1B) and negative (0, Figure 1C). We found significant variability in NDRG1 expression within and across the histological and hormone receptor subtypes of BC. NDRG1 high cases were enriched for invasive carcinoma‐no special type (IC‐NST), mixed types of carcinomas (excluding mixed ductal–lobular) and metaplastic BC, whereas mixed ductal–lobular invasive carcinomas showed the lowest proportion (Figure 1D). IC‐NST cases with low NDRG1 expression were associated with a significantly improved BCSS (Figure 1E; *p* = 0.0082). Conversely, NDRG1‐negative cases exhibited significantly reduced survival outcomes compared with NDRG1 low tumours (Figure 1E,F, *p* = 0.015). Within the tumour grades, high NDRG1 expression was enriched for Grade 3 tumours (Figure 1G). Considering survival, in Grade 1 and 2 tumours, cases with low NDRG1 expression showed significantly improved BCSS (Figure 1H), whereas no significant difference was seen in Grade 3 cases (Figure 1I). Within the hormone receptor subtypes, TNBC and HER2+ groups had higher proportions of NDRG1 high cases, whereas ER+ tumours showed an equal distribution of high and low NDRG1 expressions (Figure 1J). No survival advantage was observed for NDRG1‐low tumours in the HER2+ or TNBC group (Figure 1L,M); however, NDRG1‐low tumours in the ER+ group showed a significantly improved BCSS (Figure 1K).

**Table 1:** Clinicopathological characteristics of breast cancer tumours stratified by NDRG1 expression

	NDRG1				*p* value (Chi‐square)
	Neg (*n*, %)	Low (*n*, %)	High (*n*, %)	Total (*n*, %)	
Age (years)					
≤50	22 (22)	26 (26)	51 (52)	99 (100)	0.2977
>50	63 (29)	62 (28)	93 (43)	218 (100)	
Prognostic group					
HER2+	8 (17)	10 (21)	29 (62)	47 (100)	**0.0001**
ER+	70 (32)	74 (34)	74 (34)	216 (100)	
TNBC	10 (16)	7 (11)	45 (73)	62 (100)	
Grade					
1	19 (42)	11 (25)	15 (33)	45 (100)	**0.0001**
2	54 (35)	49 (32)	50 (33)	153 (100)	
3	18 (13)	32 (24)	86 (63)	136 (100)	
Lymph node status					
Neg	24 (30)	13 (17)	42 (53)	79 (100)	0.3808
Pos	23 (30)	19 (25)	34 (45)	76 (100)	
Size (cm)					
<2	31 (29)	34 (31)	43 (40)	108 (100)	0.2265
2–5	33 (31)	21 (19)	54 (50)	108 (100)	
>5	9 (39)	4 (17)	10 (44)	23 (100)	

Bold font indicates significant *p* values.

### NDRG1 expression correlates with progression from breast cancer to brain metastasis

**Figure 2:**
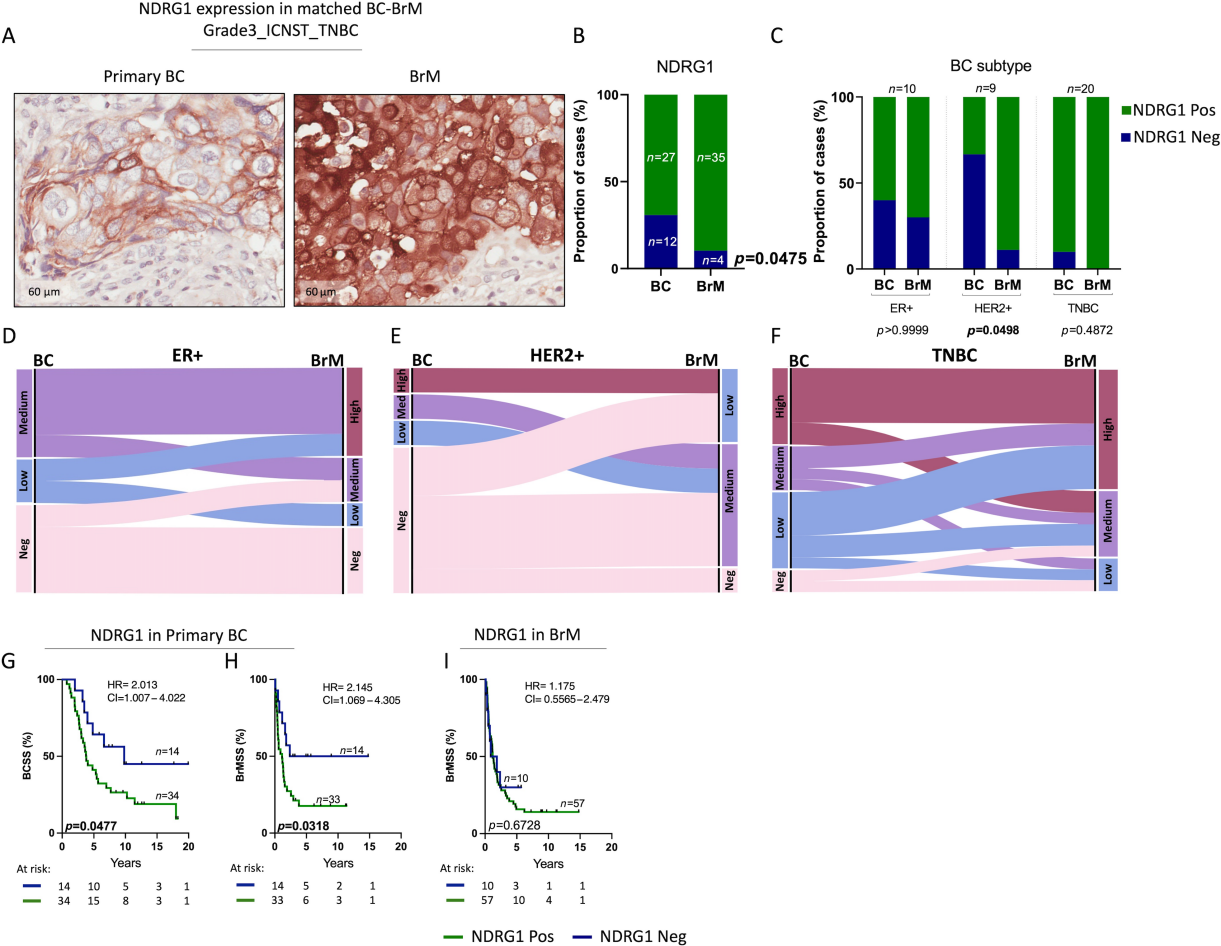
NDRG1 expression in breast cancer brain metastasis. (A) Representative IHC images of NDRG1 expression change in patient‐matched BC‐BrM sample. (B) Chi‐square analysis of proportions of matched BC and BrM cases with positive and negative NDRG1. (C) Chi‐square analysis of proportions of BC and BrM cases with NDRG1 expression compared across BC clinical subtypes. Alluvial plot representing the change in NDRG1 expression across the primary breast cancer to secondary brain metastasis stage of tumour in (D) ER+, I HER2+ and (F) TN subtypes of BC. (G) KM analysis of NDRG1 expression and BCSS in BC tumours. (H) KM analysis of NDRG1 expression and BrMSS in BC tumours. (I) KM analysis of NDRG1 expression and BrMSS in BrM tumours. BCSS, breast cancer‐specific survival; BrMSS, brain metastasis‐specific survival; ER+, oestrogen receptor; HER2+, human epidermal growth factor receptor 2; IHC, immunohistochemistry; KM, Kaplan–Meier; TNBC, triple‐negative breast cancer.

We then investigated NDRG1 expression in a cohort of BC to BrM tumours using immunohistochemistry. This cohort comprised 48 BC and 64 BrM samples, of which there were 39 patient‐matched BC‐BrM pairs. Table 2 summarises the clinicopathological characteristics of the BC‐BrM cohort based on NDRG1 expression. NDRG1 expression was first compared between the primary BC and secondary BrM tumours (Figure 2A). Broadly, NDRG1 expression was observed in a higher proportion of the BrMs compared with the primary BCs (*p* = 0.0475, Figure 2B); a significant increase was notable in the HER2+ group (*p* = 0.0498, Figure 2C). The expression pattern of NDRG1 from BC to BrM stage across each subtype is shown in Figure 2D–F. Within HER2+ cases, along with the significant gain in NDRG1 positivity, a shift from low to medium NDRG1 expression intensity was also observed (Figure 2D). Similarly, in ER+ and TNBC cases, despite no significant changes in the proportion of NDRG1‐negative cases, there was a substantial shift from the NDRG1 low and medium groups to NDRG1 high‐intensity group (Figure 2E,F). Kaplan–Meier analyses showed that NDRG1 positivity in the primary BC cases was significantly associated with poorer BCSS (*p* = 0.0477, Figure 2G) and poorer BrMSS (*p* = 0.0318, Figure 2H). No significant difference was seen in BrMSS based on NDRG1 expression in BrM (Figure 2I). BCSS and BrMSS within each subtype were also compared, and although no significant difference was observed, the trend of NDRG1 positivity and worse survival association was maintained (supplementary material, Figure S1).

**Table 2:** Clinicopathological characteristics of BC‐BrM tumours including 39 matched pairs stratified by NDRG1 localisation

		NDRG1 localisation			Total (*n*, %)	*p* value	Test
		Cyto and/or Cyto + memb (*n*, %)	Nuc and/or Cyto + memb (*n*, %)	Neg (*n*, %)			
Primary BC							
HR status	ER pos	6 (46)	1 (8)	6 (46)	13 (100)	**0.0166**	Chi‐square
	HER2 pos	3 (30)	1 (10)	6 (60)	10 (100)		
	TNBC	20 (80)	3 (12)	2 (8)	25 (100)		
	Total	29	5	14			
Grade	1/2	6 (60)	0 (0)	4 (40)	10 (100)	0.3654	Chi‐square
	3	22 (61)	5 (14)	9 (25)	36 (100)		
Time to neurosurgery (median years)		2.776	1.590	2.858		**0.0440**	Kruskal–Wallis
Brain metastases		24 (34)	37 (52)	10 (14)	71 (100)	**0.0001**	Chi‐square*

Bold font indicates significant *p* values.

* Compared to total BC samples above.

### Nuclear localisation of NDRG1 is associated with poor prognosis

**Figure 3:**
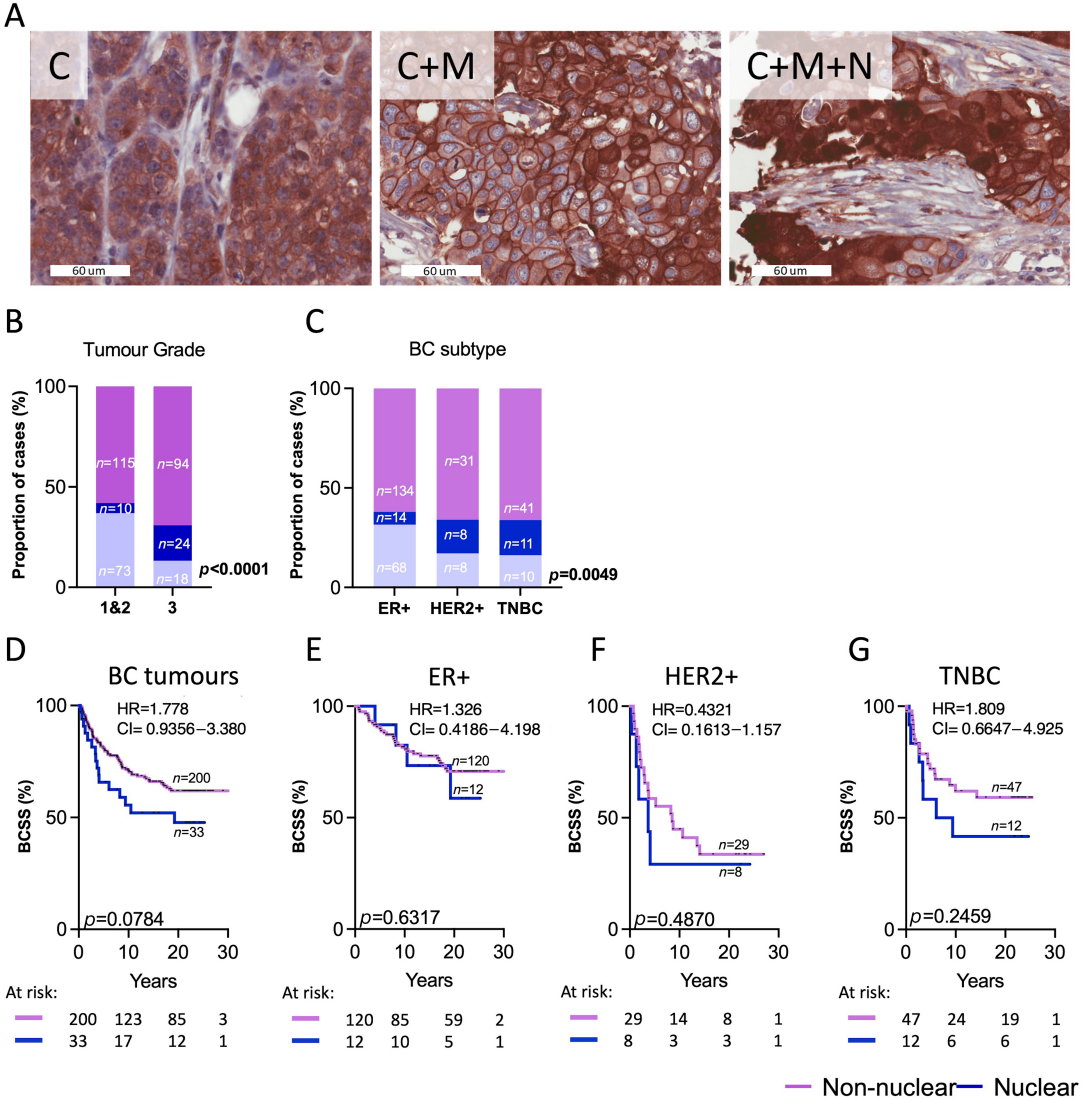
Subcellular localisation of NDRG1 in BC tumours. (A) Representative IHC images of NDRG1 localisation in BC as cytoplasmic (C), cytoplasmic + membrane (C + M) and cytoplasmic + nuclear + membrane (C + M + N). Chi‐square analysis of NDRG1 expression stratified by nuclear (C + N and/or +M) versus non‐nuclear localisations (C and/or C + M) compared across (B) tumour grade and (C) clinical BC subtypes. KM analysis of NDRG1 expression stratified by nuclear (C + N and/or +M) versus non‐nuclear localisations (C and/or C + M) and BCSS in (D) all BC tumours, (E) ER+ tumours, (F) HER2+ tumours and (G) TNBC. BCSS, breast cancer‐specific survival; ER+, oestrogen receptor; HER2+, human epidermal growth factor receptor 2; IHC, immunohistochemistry; KM, Kaplan–Meier; TNBC, triple‐negative breast cancer.

NDRG1 is localized in cellular compartments such as the plasma membrane, mitochondria and peri‐nuclear regions, which suggests diverse and potentially organelle‐specific functions [32]. NDRG1 displayed either cytoplasmic (C), cytoplasmic and membrane (C + M), or cytoplasmic, membrane and nuclear co‐localisation (C + M + N) patterns across our cohorts (Figure 3A). Nuclear NDRG1 has previously been shown to be associated with poor survival outcomes in gastric cancer [33] and has not been explored in BC or BrM. Hence, we compared the survival associations between the nuclear and non‐nuclear NDRG1 cases. The predominant phenotype in the BC cohort was non‐nuclear (C/C + M, 62.7%), with 10% of cases displaying nuclear expression (supplementary material, Figure S2). Grade 3 tumours were significantly enriched for nuclear NDRG1 expression, whereas only 5% of Grade 1 and 2 tumours showed the expression of nuclear NDRG1 (Figure 3B). Consistent with tumour grade, HER2+ and TNBC showed a higher proportion of cases with nuclear NDRG1 expression compared with ER+ cases, which consisted of 62% of non‐nuclear NDRG1 cases (Figure 3C) in BC and BC‐BrM patients. For BC patients, BCSS trended towards worse outcomes for cases with nuclear NDRG1 compared with those cases without (Figure 3D). BCSS for all three subtypes stratified by NDRG1 localisation did not show any significant changes in survival outcomes but showed similar trends for poor survival for cases with nuclear localisation (Figure 3E–G).

**Figure 4:**
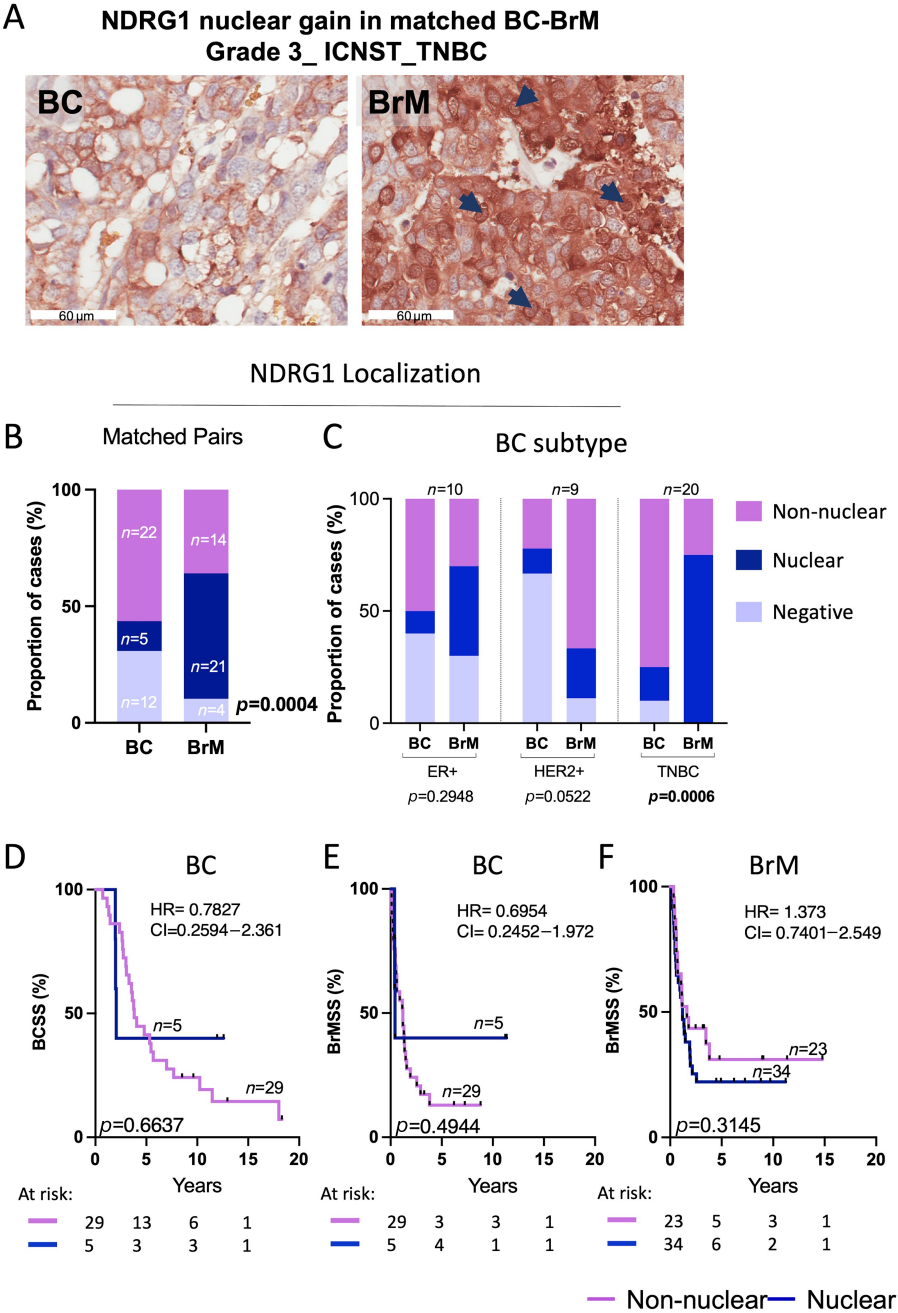
Subcellular localisation of NDRG1 in matched BC‐BrM tumours. (A) Representative IHC image of nuclear gain in BrM between matched BC‐BrM tumour cores. Nuclear NDRG1 is indicated by blue arrows in BrM tumour. (B) Chi‐square analysis of proportions of matched BC and BrM cases stratified by NDRG1 subcellular localisation. (C) Chi‐square analysis of proportions of matched BC and BrM cases for NDRG1 subcellular localisation compared across clinical BC subtypes (HER2+, ER+ and TNBC). KM analysis of NDRG1 subcellular localisation and (D) BCSS in BC tumours, (E) BrMSS in BC tumours and (F) BrMSS in BrM tumours. BCSS, breast cancer‐specific survival; BrMSS, brain metastasis‐specific survival; ER+, oestrogen receptor; HER2+, human epidermal growth factor receptor 2; IHC, immunohistology; KM, Kaplan–Meier; TNBC, triple‐negative breast cancer.

We then evaluated NDRG1 sub‐cellular localisation in the matched BC‐BrM pairs (Figure 4A) and observed a significant gain of nuclear expression in BrM compared with the original primary BC (*p* = 0.0004, Figure 4B). This nuclear gain was observed across all three clinical subtypes with the most striking gain of nuclear expression in the TNBCs (Figure 4C). In primary BC, no significant differences in BCSS were observed (Figure 4D) likely because of the low numbers expressing NDRG1 in the nucleus. Equally, nuclear NDRG1 in primary BC tumours did not show any significant trend towards BrMSS, although non‐nuclear localisation is significantly associated with worse BCSS (*p* = 0.0210, Figure 4E). Nuclear NDRG1 in BrM tumours did not show survival differences (Figure 4F), but given BrMSS is uniformly poor, it is challenging to significantly stratify further within this group. We also compared C and C + M localisations of NDRG1 with survival outcomes and saw a significant association of C + M NDRG1 expression with worse survival outcomes in BC patients, especially in ER+ patients. In BrM patients, neither nuclear nor C + M localisation showed survival associations (supplementary material, Figure S2).

We then considered the prognostic value of the NDRG1 expression and localisation status compared with standard clinicopathology variables within the QFU dataset. As shown in Table 3, in a multivariate analysis, NDRG1 expression status adds significant prognostic value, with a lack of expression [hazard ratio (HR) = 2.33; *p* = 1.48E‐03] adding greater prognostic value than ER positivity (HR = 0.62; *p* = 1.03E‐01). While NDRG1 localisation was prognostic (HR = 1.36; *p* = 2.85E‐01), an absence of staining contributed more value in the current dataset (HR = 1.99; *p* = 1.20E‐03). Notably, HER2 status was the most prognostic variable, reflecting the historical nature of this cohort and it being pre‐HER2‐targetted therapy implementation.

**Table 3:** Multivariate analysis of clinicopathologic variables and NDRG1

	Variable	Variable test	Value	HR	Lower CI	Upper CI	*p* value
NDRG1 expression	ER	ER: Pos	Pos	0.62	0.35	1.10	1.03E−01
	PR	PR: Pos	Pos	0.81	0.48	1.37	4.25E−01
	Age	Age: ≥50	≥50	0.90	0.62	1.32	5.97E−01
	NDRG1 status	NDRG1: High	High	1.39	0.83	2.32	2.10E−01
	NDRG1 status	NDRG1: Neg	Neg	2.33	1.38	3.94	1.48E−03
	HER2	HER2: Pos	Pos	2.78	1.77	4.35	8.23E−06
NDRG1 localisation	ER	ER: Pos	Pos	0.58	0.33	1.01	5.43E−02
	PR	PR: Pos	Pos	0.80	0.47	1.35	4.03E−01
	Age	Age: ≥50	≥50	0.92	0.63	1.34	6.58E−01
	NDRG1 localisation	NDRG1: Cyto + Memb and/or Nuc	Cyto + Memb and/or Nuc	1.36	0.77	2.41	2.85E−01
	NDRG1 localisation	NDRG1: Neg	Neg	1.99	1.31	3.03	1.20E−03
	HER2	HER2: Pos	Pos	2.79	1.78	4.39	8.27E−06

### NDRG1 expression is associated with *NDRG1* amplification

**Figure 5:**
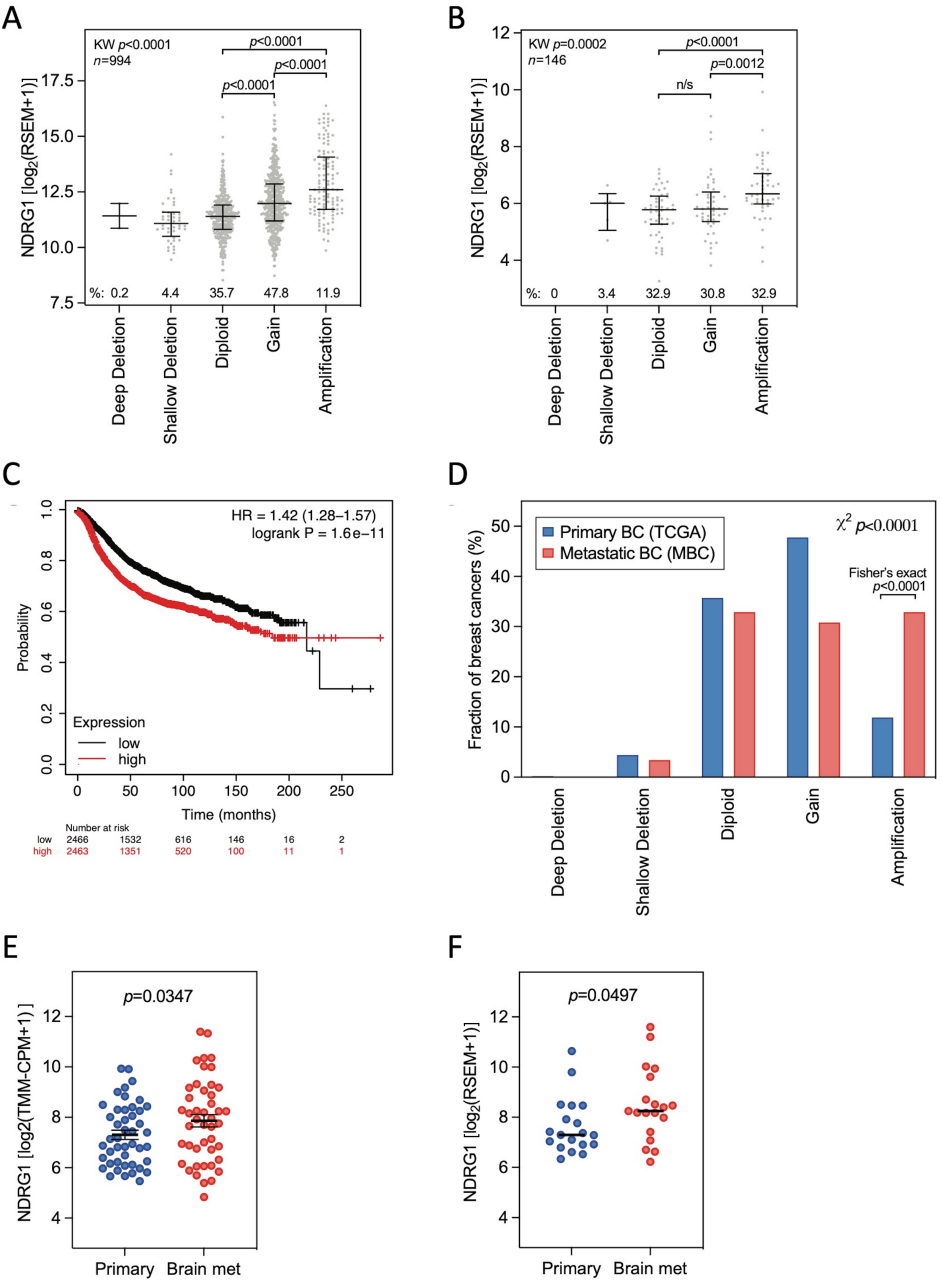
NDRG1 expression is associated with *NDRG1* amplification, poor prognosis and brain metastasis in breast cancer. NDRG1 expression levels according to *NDRG1* copy number status using The Cancer Genome Atlas (TCGA) dataset (A) and the Metastatic Breast Cancer (MBC) project dataset (B). *p* values: Kruskal–Wallis tests (KW) and Mann–Whitney *U* tests. (C) Recurrence‐free survival analysis of breast cancer patients (*n* = 4,929) using the median NDRG1 expression as the cut‐off between patients expressing low and high NDRG1 levels. HR, hazard ratio. (D) Distribution of fractions of patients with respective *NDRG1* copy number statuses in primary and metastatic breast cancers in the TCGA and MBC datasets. (E, F) NDRG1 expression levels in primary and metastatic breast cancers in 45 matched pairs (E) and 38 independent samples (F). *p* values: paired Student *t* test and Mann–Whitney *U* test, respectively.

Given the association of over‐expression with poor survival, we considered that there may be a molecular origin for the increased NDRG1 expression. *NDRG1* is located on chromosome arm 8q, which is frequently gained in primary and metastatic BCs [34]. In two independent datasets, we found that *NDRG1* is frequently gained (three copies) or amplified (greater than three copies) in primary and metastatic BCs (59.7% and 63.7%, respectively; Kruskal–Wallis *p* < 0.0001; Figure 5A) and that this is also associated with increased NDRG1 expression (Figure 5B; Kruskal–Wallis *p* = 0.0002, respectively). Additionally, consistent with previous work in inflammatory BC [12], pooled primary BCs [35] and our protein data, *NDRG1* expression is associated with poor prognosis in BC (Figure 5C).

We then investigated *NDRG1* copy number status and expression in primary BCs and BC BrMs. First, *NDRG1* is more frequently amplified in BrMs than in primary BCs (Figure 5D; *p* < 0.0001). Second, in two independent datasets, *NDRG1* expression is significantly higher in BrMs than in primary BCs, both in a paired analysis of 45 primary‐metastatic cancer pairs (*p* = 0.0347; paired Student *t* test) (Figure 5E) and in an unpaired analysis of 90 samples (*p* = 0.0497; Mann–Whitney *U* test) (Figure 5F). Thus, *NDRG1* amplification and increased expression are associated with BC BrM.

### Hypoxic stress upregulates NDRG1 expression and subcellular localisation

**Figure 6:**
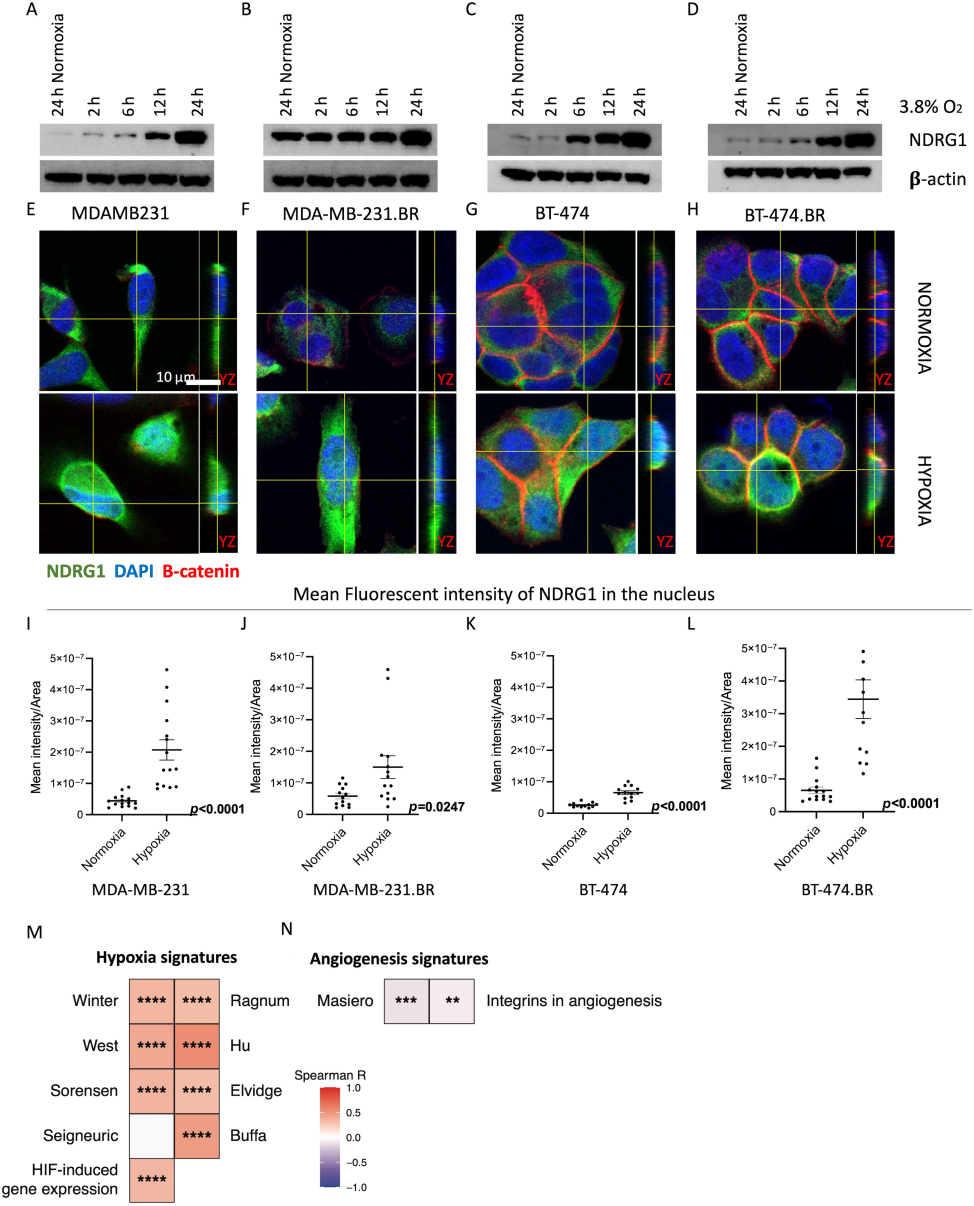
Change in NDRG1 expression under hypoxia. Representative immunoblots of NDRG1 protein expression in (A) MDAMB231, (B) MDAMB231 BR, (C) BT474 and (D) BT474 BR cells cultured under hypoxia. Confocal microscopy images of NDRG1 and Beta‐catenin expression in (E) MDAMB231, (F) MDAMB231 BR, (G) BT474 and (H) BT474 BR cells cultured under normoxia (upper panel) and hypoxia (lower panel). Schematic representation of quantification analysis method using ZEN 3.5 lite for confocal images. Quantification of nuclear NDRG1 expression in (I) MDAMB231, (J) MDAMB231 BR, (K) BT474 and (L) BT474 BR cells cultured under hypoxic and normoxic conditions. Single‐sample gene set enrichment analysis (ssGSEA) scores for (M) hypoxia signatures and (N) angiogenesis signatures compared with *NDRG1* expression using RNAseq data from breast cancers in the TCGA dataset. Colour scale: Spearman correlation *R*. Spearman *p* values: ****p < 0.0001; ***p < 0.001; **p < 0.01.

We considered what might drive the localisation of NDRG1 in BC and BrMs. NDRG1 relocates to the trophoblast nucleus under hypoxic stress [36], and hypoxia is one of the major hallmarks of cancer. Tumours of the brain often create an hypoxic environment. Thus, to determine whether hypoxic stress was influencing NDRG1 localisation in BC and its metastases, we cultured MDAMB231 (TNBC) and BT474 (HER2+) and their brain metastatic derivatives (.Br) in hypoxic conditions (3.8% O_2_) over a 24‐h time course. An overall increase in NDRG1 expression was observed across all four cell lines in a time‐dependent manner under hypoxic conditions compared with cells incubated under normoxia (19% O_2_; Figure 6A–D). This was confirmed in three independent experiments, with a significant increase at the 24‐h time point (supplementary material, Figure S3).

It has also been reported that NDRG1 can modulate the nuclear translocation of beta‐catenin and hence influence epithelial‐to‐mesenchymal transition in prostate and colon cancer cells [37]. To investigate whether NDRG1 subcellular localisation correlates with beta‐catenin localisation, confocal microscopy was used. Consistent with the immunoblots, we observed that the overall NDRG1 expression was higher in cells grown under hypoxic conditions, with enrichment particularly in the cytoplasm (Figure 6E–H). Z‐stack images were used to assess this localisation in the YZ plane of all four cell lines. With respect to beta‐catenin, as expected, TNBC cells (MDA‐MB‐231) exhibited low and scattered expression, whereas strong membrane staining was evident in HER2+ cells (BT‐474). No change in beta‐catenin localisation or expression was found in cells between normoxic and hypoxic conditions. Quantification of nuclear NDRG1 expression was performed using the Zen3.5 Lite software, and the mean values of multiple ROIs (*n* = 14) were calculated. A significant gain in nuclear NDRG1 under hypoxia was observed for all cell lines (Figure 6I–L). To assess whether this association may also exist in an independent cohort of BC patients, we determined hypoxia and angiogenesis gene expression scores in BC samples from TCGA and compared these with the *NDRG1* expression levels. This revealed that eight of nine hypoxia signatures showed highly statistically significant positive associations with *NDRG1* mRNA (Spearman *p* < 0.0001) (Figure 6M). Conversely, both angiogenesis signatures correlated negatively with *NDRG1* expression (Spearman *p* < 0.01) (Figure 6N). Thus, these data suggest that *NDRG1* expression also increases under hypoxic conditions in BC patients.

## Discussion

Although NDRG1 is established as a metastasis suppressor [38], increasing evidence demonstrating its pro‐tumorigenic and pro‐metastatic roles [39, 40, 41] has raised intriguing questions about its biology. Berghoff *et al* recently reported the involvement of NDRG1 in BC BrM progression using *in vitro* and *in vivo* models. They reported high NDRG1 expression in slow‐cycling BC cells involved in the development of BrMs and further showed that downregulating NDRG1 resulted in a complete suppression of BrM development [42]. Villodre *et al* investigated NDRG1 in both inflammatory BC [12] and BC BrM [13], and reported that NDRG1 expression was significantly higher in ER‐negative and Grade 3 BC. They also showed depleting NDRG1 significantly reduced the colony forming, migratory and invasive properties of BC cells including the capacity of BC cells to establish BrMs.

Our clinicopathological analysis of NDRG1 corroborates and extends these studies. We examined a cohort of matched primary BC BrM tumours as well as a cohort of unselected BC and found NDRG1 expression in the majority of tumours in both the cohorts, further supporting NDRG1's role as an oncoprotein [15]. Conversely, a complete absence of NDRG1 also demonstrated a significant association with poor survival, arguing for its role as a tumour suppressor. Such a ‘Goldilocks’ or ‘sweet‐spot’ protein dosage is not a new concept in broader signalling pathway research but is certainly the one that can account for the previous inconsistent findings for NDRG1 across different cancer types [43, 44, 45].

Considering the high levels of NDRG1 expression, we observed a significant association with worse BCSS in our two independent cohorts, demonstrating the potential of NDRG1 as a prognostic indicator in BC. Within the matched BC‐BrM patient cohort, NDRG1 positivity was higher at the BrM stage of tumour progression. This NDRG1 gain at the BrM stage was maintained across all three BC clinical subtypes in matched cases, with the most significant shift in HER2+ cases. Unlike HER2+ cases, ER+ cases did not exhibit a shift from negative to positive NDRG1 expression at the BrM stage, but an overall gain of NDRG1 expression was still evident. In TNBC, 90% of cases were positive for NDRG1 in the primary BC itself, but the distinction observed at the BrM stage was the transition into stronger NDRG1 expression from medium and low NDRG1 cases. Considering the matched pairs of primaries to BrMs, it was clear that NDRG1 expression is activated at some point during either colonisation or expansion in the brain, with an increase in positivity and intensity shown. NDRG1 expression across tumour grades also indicated an association of NDRG1 high expression with higher tumour grade. Additionally, HER2+ and TNBC cases contained a much higher proportion of NDRG1 high cases, whereas ER+ tumours had an equal proportion of NDRG1 high and low expressions. NDRG1 high expression in both HER2+ and TNBC subtypes is likely reflective of the association of NDRG1 expression with more aggressive subtypes of BC, also confirming the work of Villodre *et al* [12].

NDRG1 expression in the primary BC was significantly associated with poorer BrMSS. NDRG1 has been shown to exhibit variability in its function depending on the tumour type or cell type it is expressed in. Our findings demonstrate that high NDRG1, as well as no NDRG1 expression, could potentially lead to the same clinical outcomes of poorer prognosis in BC, hinting at another layer of context‐dependent complexity.

At the subcellular level, NDRG1 shows a variable localisation pattern, primarily concentrated in the cytoplasm, while also being detected in the plasma membrane and to a lesser extent, the nucleus [32]. NDRG1 localisation in hepatocellular carcinoma, and prostate, colon and pancreatic cancer has been shown to have functional implications [32, 46, 47]. Work by Villodore *et al* and Berghoff *et al* noted cytoplasmic and membranous NDRG1 staining, but the relationship with patient prognosis has not yet been reported in BC. Nuclear expression of NDRG1 was significantly enriched for Grade 3 tumours in the BC patient cohort, as well as specifically in the HER2+ and TNBC cases. Nuclear NDRG1 expression trended towards poor BCSS, and this trend was seen across both HER2+ and TNBC subtypes. Few BC cases exhibited nuclear NDRG1 expression, similar to what was seen in the primary BC of the BC‐BrM cohort, and within the matched pairs, a significant gain of nuclear NDRG1 expression was evident at the BrM stage. Localisation changes were compared across BC and BrM and revealed that HER2+ cases were more likely to gain cytoplasmic and membranous NDRG1 expressions, whereas ER+ and TNBC tumours showed a nuclear gain. Interestingly, 80% of TNBC cases switched to a gain of nuclear expression at the BrM stage, indicating that even though most of the TNBC tumours were positive for NDRG1 expression in both BC and BrM, there was a clear distinction in the localisation of NDRG1 expression. BrMSS outcomes are limited because of the poor clinical journey of BrM patients; however, BrM cases with nuclear expression of NDRG1 still trended towards the worst BrMSS, indicating the nuclear NDRG1 association with BrM. Furthermore, our multivariate analysis shows that NDRG1 expression (either high or absent) and localisation were second only to HER2 status as prognostic variables of value.

NDRG1 is a stress‐responsive protein activated under hypoxia, a central hallmark of cancer progression [48, 49, 50]. While NDRG1 has been shown to translocate to the nucleus under hypoxic influence in trophoblasts [36], no such response has been reported in the context of BC or its metastasis. Considering that HER2+ and TNBC tumours showed a greater enrichment overall for NDRG1, we studied BT474 and MDAMB231 cell lines in a hypoxic environment. Total NDRG1 expression was found to be significantly increased under hypoxia for multiple time points in both BT474 and MDAMB231 as well as in their brain‐seeking derivative cells, and we show concomitant nuclear enrichment. Interestingly, nuclear enrichment of NDRG1 was not observed in a similar hypoxic stress study in hepatocellular carcinoma [51]. We also identified a strong positive correlation between NDRG1 expression and hypoxia signatures in BC samples, confirming the close association between NDRG1 and hypoxia. Additionally, NDRG1 expression was found to negatively correlate with an angiogenesis signature, another hallmark process in cancer [52].

The exact function of NDRG1 in the nucleus remains unclear, and the lack of a nuclear localisation signal in the NDRG1 protein sequence is curious. Park *et al* previously discussed two isoforms of NDRG1: full length (FL) and truncated (T), in prostate and pancreatic cancer cells [32]. FL NDRG1 and phosphorylated NDRG1 (Ser330) were localized in the nucleus, whereas the T NDRG1 was detected in the cytoplasm only. Contrary to these findings, Shi *et al*, in their work in trophoblasts [36], showed that deletion of the N‐terminus region of NDRG1 did not influence the nuclear localisation of NDRG1 under hypoxia. Our study used a C‐terminus antibody (ab124689), which was in agreement with the Park *et al* work, and we detected both cytoplasmic and nuclear NDRG1. It is evident from our data that nuclear NDRG1 is an important consideration moving forward to further understand NDRG1's role in breast and other cancer progression. The association between nuclear staining and poorer outcome in our study may relate to the enhanced level of cellular stressors like genomic instability and hypoxia in aggressive tumours and metastases [53], but it is evident that more detailed investigation of nuclear NDRG1 is essential.

Taken together, our data confirm that both high NDRG1 protein expression and an absence of NDRG1 correlate with poorer outcomes in BC and also in BrM. We thus consider NDRG1 to be a ‘Goldilocks’ cancer protein, where too much or too little has a significant impact on survival. We show that nuclear localisation of NDRG1 is the prominent phenotype in BrMs and that hypoxic conditions promote an increase in expression and nuclear localisation. The function of NDRG1 in BC and its metastases remains elusive but an intriguing prospect for future studies.

## Supporting information


**Figure S1.** NDRG1 expression in breast cancer brain metastasis
**Figure S2.** KM analysis of NDRG1 subcellular localization and BCSS stratified by clinical subtype (E) ER+, (F) HER2+, and (G) TNBC
**Figure S3.** Change in NDRG1 expression under hypoxia

## Data Availability

The data that support the findings of this study are available from the corresponding author upon reasonable request.
