# Supplementary material for: NDRG1 is a prognostic biomarker in breast cancer and breast cancer brain metastasis
Source: J Pathol Clin Res. 2024 Feb 5;10(2):e12364. doi: 10.1002/2056-4538.12364 (PMC10839626; doi:10.1002/2056-4538.12364)
Supplement: Supplementary file 1 — Figure S1. NDRG1 expression in breast cancer brain metastasis Figure S2. KM analysis of NDRG1 subcellular localization and BCSS stratified by clinical subtype (E) ER+, (F) HER2+, and (G) TNBC Figure S3. Change in NDRG1 expression under hypoxia [file CJP2-10-e12364-s001.pdf]

NDRG1 is a prognostic biomarker in breast cancer and breast cancer brain metastasis

V Joshi *et al*., *J Pathol Clin Res*, https://doi.org/10.1002/2056-4538.12364

Supplementary Figures S1–S3

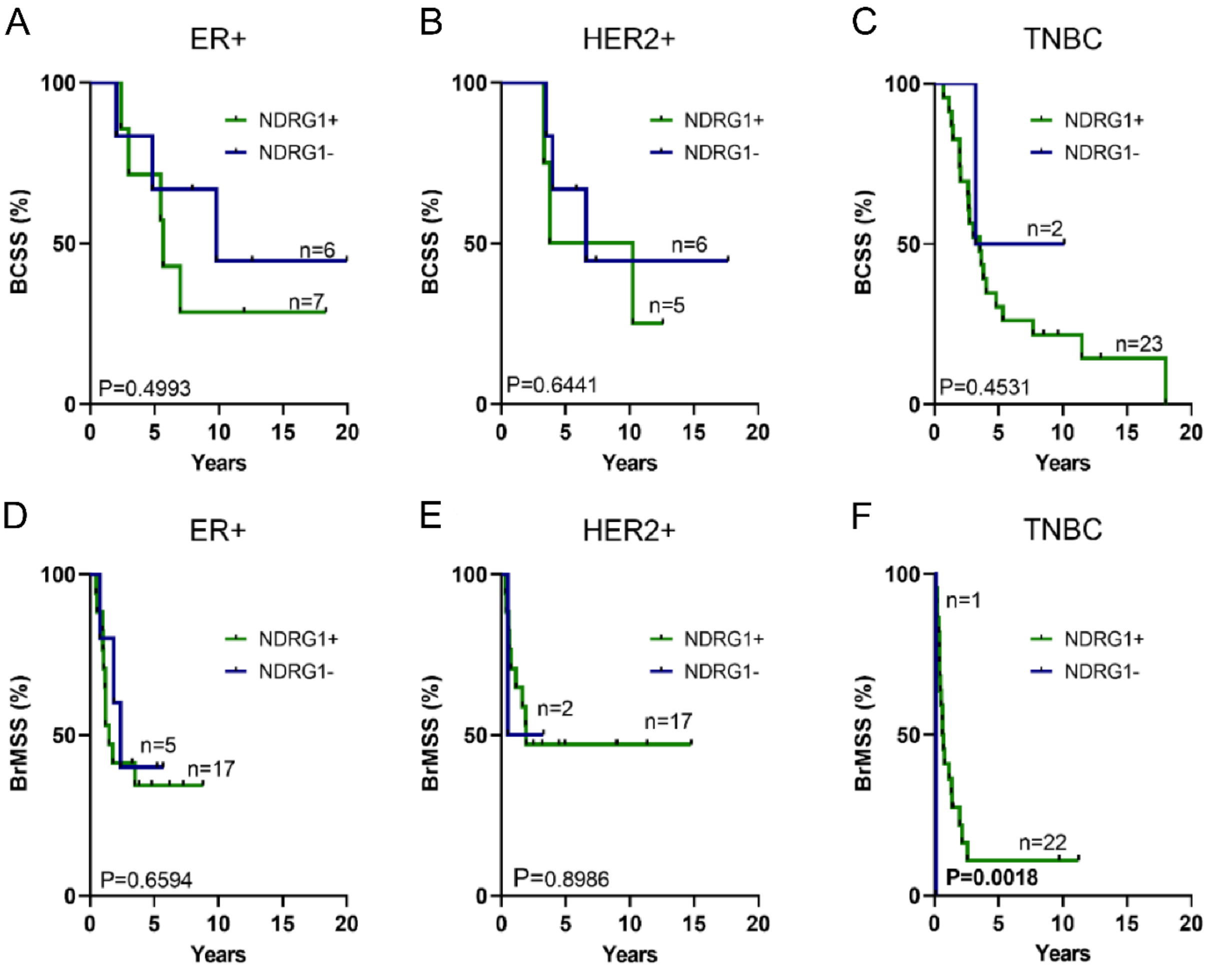

Figure S1. NDRG1 expression in breast cancer brain metastasis. KM analysis of NDRG1 expression in BC tumours and BCSS in **(A)** ER+ **(B)** HER2+ , **(C)** TNBC BC subtypes. KM analysis of NDRG1 expression in BrM tumours and BrMSS in **(D)** ER+, **(E)** HER2+, **(F)** TNBC subtypes.

BCSS, Breast cancer specific survival; BrMSS, Brain metastasis specific survival; ER+, oestrogen receptor; HER2+, Human epidermal growth factor receptor 2; KM, Kaplan Meier; TNBC, triple negative breast cancer.

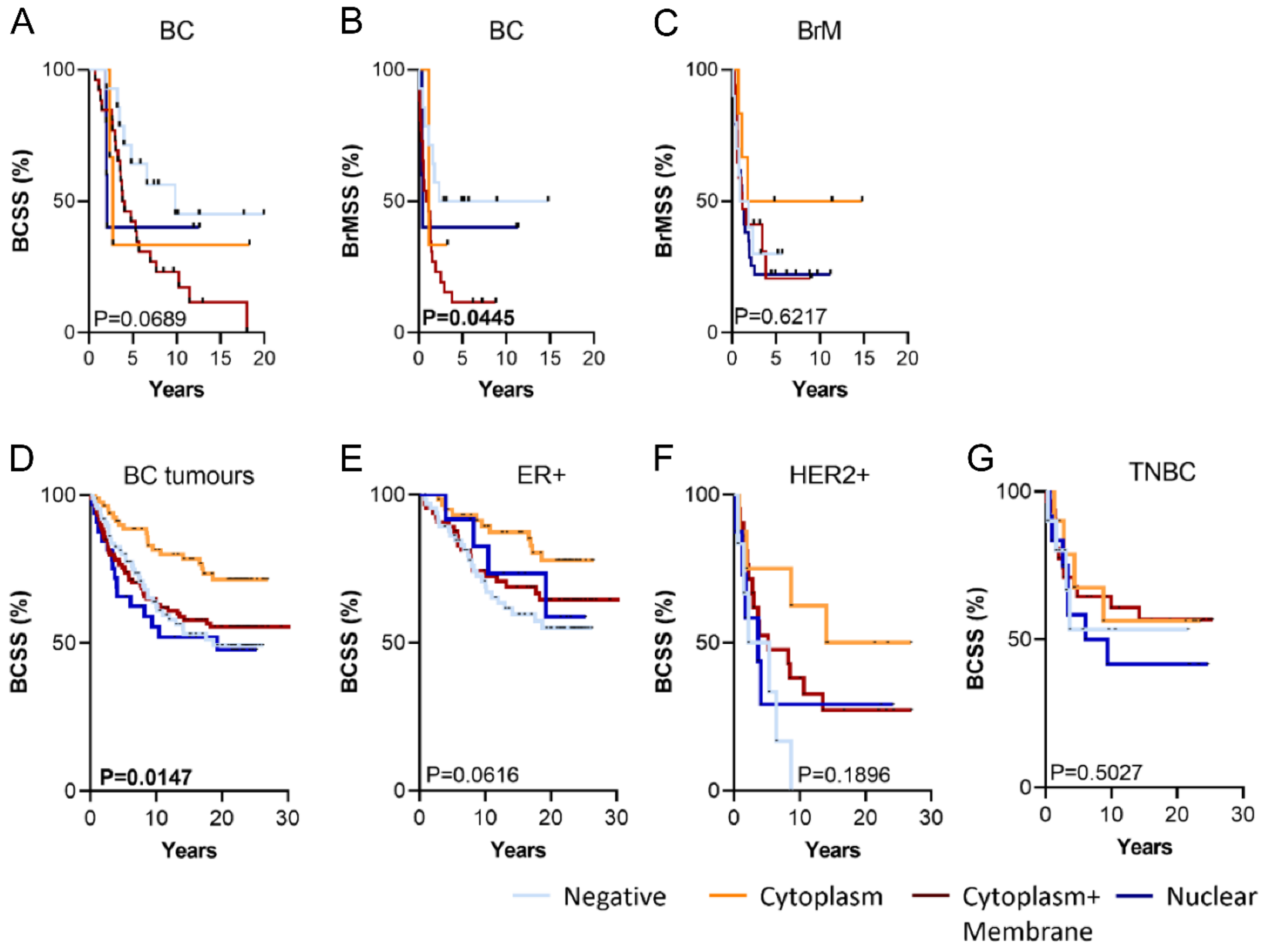

Figure S2. NDRG1 localisation in breast cancer and breast cancer brain metastasis. KM analysis of NDRG1 subcellular localisation and **(A)** BCSS in BC tumours, **(B)** BrMSS in BC tumours, and **(C)** BrMSS in BrM tumours. **(D)** KM analysis of NDRG1 subcellular localization and BCSS in all BC tumours. **(E-G)** KM analysis of NDRG1 subcellular localization and BCSS stratified by clinical subtype: **(E)** ER+, **(F)** HER2+, and **(G)** TNBC.

BCSS, Breast cancer specific survival; BrMSS, Brain metastasis specific survival; KM, Kaplan Meir; ER+, oestrogen receptor; HER2+, Human epidermal growth factor receptor 2; TNBC, triple negative breast cancer.

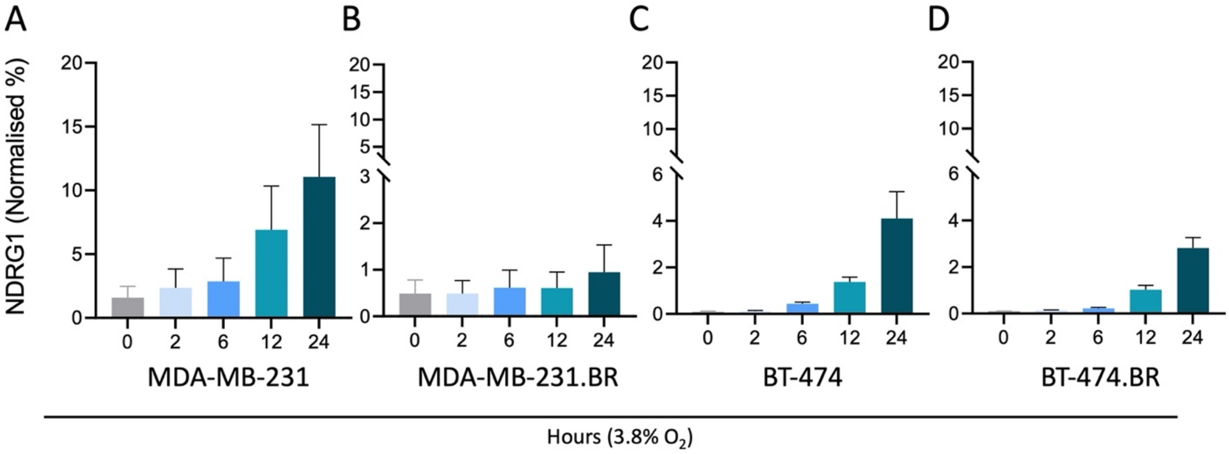

Figure S3. Change in NDRG1 expression under hypoxia. Quantified analysis of change in NDRG1 protein expression in **(A)** MDA-MB-231, **(B)** MDA-MB-231.BR, **(C)** BT-474, **(D)** BT-474.BR cells.
